# Supplementary material for: Exploring the acute affective responses to resistance training: A comparison of the predetermined and the estimated repetitions to failure approaches
Source: PLoS One. 2021 Aug 18;16(8):e0256231. doi: 10.1371/journal.pone.0256231 (PMC8372906; doi:10.1371/journal.pone.0256231)
Supplement: Attachment — Submitted filename: Reviewer_PlosOne (1).docx [file pone.0256231.s002.docx]

**Reviewer #: General article comments:**

**Comment:**

This study examined the effects of two-volume training prescription approaches during resistance training on affective responses. **I like the specific theme of the paper and the results presented within it.** The weakness is probably in the description of the methods, for the most part. It would be difficult for other researchers to replicate these methods. **Please be aware that all my comments and suggestions presented here are in order to improve the article.**

Response: We thank the reviewer for the favorable response. We address the comments point by point below.

**Please see my specific comments:**

**Ln23-29. The purpose of the study has not been described in the abstract section.**

Response: We thank the reviewer for bringing this point to out attention. We now describe the purposes of the study in the abstract section. (lines: 26-28). Note that we have also changed the title of the manuscript to emphasize the primary aim of the study.

**Ln 31. what exercises?**

Response: We have now added the names of the exercises to the abstract section. (lines: 39-41).

**Ln 39. Exercises should be described in the Methods section**

Response: In response to the previous comment, we inserted exercises names to the methods section of the abstract (lines: 39-41).

**Ln 48. Generally speaking, Introduction is well-written**

Response: We thank the reviewer for this comment.

**Ln 62. it is not intensity (%1RM), but the level of effort (i.e. relationship between repetitions performed and repetitions that could be completed (Sanchez-Medina, L, and Gonzalez-Badillo, JJ. Velocity loss as an indicator of neuromuscular fatigue during resistance training. *Med Sci Sports Exerc* 43: 1725-1734, 2011)**

Response: We have modified this part of the introduction to better conveys the idea that reaching different proximities to task failure is associated with different levels of relative effort and may lead to training outcomes (lines: 63-65). Note that this concept is also elaborated on in paragraph #2 (lines: 70-73).

**Ln 70. Please use a reference for this statement.**

Response: We have changed the paragraph and this sentence no longer appears at this form. Note that a more speculative version of this idea appears in line 65.

**Ln 108. why did you use inexperienced subjects? Are they familiarized with repetitions to failure? and with the feeling of how many repetitions in reserve they leave?**

Response: We have now added a detailed explanation for selecting the study's cohort. (lines: 126-129).

**Material and Methods**

**This section could be improved qualitatively adding some detailed explanations about the research process. Please attend to specifics comments below.**

**Ln 119. if we don´t know how many repetitions they could perform. How can we know the bias in their estimation?**

Response: We thank the reviewer for this comment. We would like to draw the reviewer's attention to the prediction accuracy of the ERF scales across experimental cohorts. Both Hackett and Zourdos examined several cohorts where both experienced and inexperienced trainees accurately predicted repetitions to failure (see references 14,15,24,25,26 in the manuscript). We discuss the evidence regarding prediction accuracy in the discussion section of the manuscript (lines 295-297). We also acknowledge that not reaching actual failure during our investigation is a weakness of the study design. We discuss it in the limitations segment of the discussion (lines 304-310).

**Ln 122. how long was the recovery period between sessions? Did you control how they felt before the sessions? What they performed the previous day? Did you control the presence of a coach or partners? Or even music.**

Response:

1. The recovery period is described in the methods section. We ensured a minimum of three days interval between sessions. The mean interval was 6.2 days, and the range was 3-14 days. This information appears in the manuscript (lines150-151).
2. Participants rated their affective valence via the Feeling Scale before the first set of the first exercise. They did not rate it upon arrival. We were interested in the acute influence of repetitions-prescription, therefore we decided that the relevant information is how they feel before and after the set.
3. We asked participants to refrain from strenuous physical activity on the day before their sessions. This information appears in lines 162-163.
4. This study was conducted at the University gym. This ecological setting did not allow us to control for the presence of other trainees. We did schedule the sessions at approximately the same time of day (line 164), so that the capacity of trainees at the gym was approximately similar for each participant.

**Ln 136. Table 1. Please use the same number of decimals in mean and SD**

Response: We thank the reviewer for drawing our attention to this. We have corrected the decimals (line 140).

**Ln 147. what kind of leg-press? 45º? 60º? weight stack machine?**

Response: We have now specified the type of equipment and version of the exercises (line 152).

**Ln 153. Since they were novices in RT, did you perform familiarization sessions? Did you standardized hand and feet positions? How did you control technique execution? How much rest between exercises?**

Response: We did not perform a familiarization session but chose to use a 5RM prediction procedure instead – allowing novice trainees a fair estimation of their 1RM without reaching intimidating levels of effort (line 190-193). We acknowledge this is a limitation of the study design and discussed in the limitations section of the discussion (lines 316-318). Regarding the position and technique, we now added more details to this segment of the methods section (lines156-162).

**Ln 217. Is correction for multiple comparisons necessary? I think that you only have 2 time-points**

Response: Since we compared several outcomes that have some dependencies, correcting for multiple comparisons is recommended to reduce the likelihood of the type 1 error.

**RESULTS
Table 2. CI of effect size in the FS parameter. Shouldn´t they be symmetric? Or I guess that one decimal number in the mean is missing.**

**please use the same number of decimals for ES and CI**

Response: We thank the reviewer for the attention to details. This is a matter of decimals. We did not find it more informative to include more than two decimals. Following the reviewer's comments, we corrected all tables so that number of decimals is similar.

**DISCUSSION
Although Discussion is generally well-written, only 4 references have been used (in lines 266-267). Please use previous literature to compare and discuss your findings.**

Response: We thank the reviewer for this comment. We now cite more references throughout the discussion section (e.g., lines 297-299).

**Ln 301. why not beginners in order to increase their adherence?**

Response: We now added an explanation regarding our selection of cohort (line 126-129). We also discuss the possible limitations of this cohort in the discussion section (lines 312-316).

**Ln 302. Doing a 5RM test of 4 exercises without experience and even without familiarization is another limitation. May influence the fatigue of the first exercise on the 1RM estimation in the following exercises?**

Response: We thank the reviewer for this comment. We allowed three minutes of rest after the last set of each 5RM test before the next exercise was introduced. Then, the participant performed two sets with light weight before beginning to increase weight to reach the next 5RM set. This process allowed for a minimum of ten minutes between maximal sets of each exercise.

**Figure. These two outliers in LP should be discussed**

Response: The outliers are now discussed (lines 320-323).

**Reviewer #: Thank you for inviting me to review the article "Repetition numbers in resistance training: a comparison between the predetermined and the estimated repetitions to failure prescription approaches on affective responses"
in my view, regrettably, this paper does not meet the eligibility requirements for publication in this journal.
This rejection is related to a combination of reasons, including: the experimental impact, impact and importance in the field of strength and conditioning, the poor sophistication of the study and the experimental methods used. Moreover, no innovative information is brought to the existing scientific literature and the research question is not very clear.**

**In addition, performing a 5RM test (to estimate 1RM) in subjects with no previous experience in RT, without any familiarization session and evaluating the effects of a single session carried out with the protocols do not allow to have clear, exhaustive and generalizable results.**

Response: We thank the reviewer for taking the time to review our work. Unfortunately, after a careful examination of the comments and concerns brought up by the reviewer, we found the reasons for rejecting the manuscript unjustified and unclear.  The reviewer heavily emphasized aspects that are not relevant for PLOS ONE rejection, since novelty, innovation, and impact, are very subjective and debatable and do not concern methodology**.** The only clear criticism the reviewer brought up concerns our choice to implement 5RM tests with untrained participants. Using the 5RM prediction procedure and Brzycki's equation with this cohort was based on literature confirming the accuracy of this equation combined with our reluctancy to introduce intimidating procedures such as the 1RM test. This information appears in the methods section of the manuscript (lines 191-193). It is also acknowledged as a possible limitation in the last segment of the discussion (lines 316-323).
